# Anticipatory prediction of sit-to-stand and stand-to-sit transitions: a unified approach

**DOI:** 10.3389/fbioe.2026.1792582

**Published:** 2026-04-01

**Authors:** Kai Ren, Yuichi Nakamura, Kazuaki Kondo, Kei Shimonishi, Qi An, Takahide Ito, Jun-ichiro Furukawa, Masashi Toda, Junichi Akita

**Affiliations:** 1 Nakamura Laboratory, Graduate School of Informatics, Kyoto University, Kyoto, Japan; 2 Academic Center for Computing and Media Studies, Kyoto University, Kyoto, Japan; 3 Department of Human and Engineered Environmental Studies, Graduate School of Frontier Sciences, The University of Tokyo, Tokyo, Japan; 4 Guardian Robot Project, RIKEN, Kyoto, Japan; 5 Faculty of Systems Engineering, Wakayama University, Wakayama, Japan; 6 Center for Management of Information Technologies, Research and Education Institute for Semiconductors and Informatics, Kumamoto University, Kumamoto, Japan; 7 College of Transdisciplinary Sciences for Innovation, Kanazawa University, Kanazawa, Japan

**Keywords:** momentum transfer strategy, muscle synergies, stabilization strategy, stand-to-sit transition, surface electromyography

## Abstract

**Introduction:**

Sit-to-stand (*S_i_TS_t_*) and stand-to-sit (*S_t_TS_i_*) motions, collectively referred to as STS motions, are fundamental movements for independent daily living. However, many individuals are unable to generate sufficient strength and balance during these motions, which increases the risk of fall-related accidents. Therefore, proper and timely mechanical assistance is needed to improve the quality of daily life for people with weak muscles or insufficient motor control.

**Methods:**

To support the development of such assistance, we analyzed *S_i_TS_t_* and *S_t_TS_i_*) motions performed using two different strategies by means of electromyography. Muscle synergy analysis was used to provide a compact and physiologically interpretable description of myoelectric patterns, enabling systematic comparisons of neuromuscular control across the two movement strategies, namely the momentum transfer strategy and the stabilization strategy. Based on these findings, we further proposed a deep neural network framework to predict motion states prior to motion initiation.

**Results:**

The experimental results demonstrated that at least four synergy patterns were sufficient to represent these STS motions. In addition, the proposed method achieved an accuracy of 92.97 ± 0.86% with a forecasting time of 300 ms for motion state prediction, while the average temporal error remained consistently below 50 ms.

**Discussion:**

These findings indicate that muscle synergy analysis can effectively characterize different STS movement strategies and that the proposed deep neural network framework can provide sufficient lead time for assistive device activation. This approach may contribute to the development of effective mechanical assistance systems for individuals with impaired muscle strength or motor control.

## 1 Introduction

Sit-to-stand $\left(S_{i}TS_{t}\right)$ and stand-to-sit $\left(S_{t}TS_{i}\right)$ motions are important for daily activities, both of which are essential for maintaining personal mobility and independent functioning. Healthy people execute both STS motions considerably; a previous study showed that each person repeats $S_{i}TS_{t}$ motions approximately 33–71 times per day (Bohannon, 2015). Despite this importance, people often fail to generate sufficient strength and balance, which readily leads to fall-related accidents. Thus, there is substantial demand for mechanical assistance for these motions, which may continuously improve the quality of daily life of people with weak muscles or insufficient muscle control ability. In addition, such assistive systems may contribute to rehabilitation by promoting muscle activation, thereby supporting neuromuscular recovery in a controlled and safe environment.

Thus, numerous studies have extensively explored automatic assistance for $S_{i}TS_{t}$ movements; e.g., (Li et al., 2016; Martinez-Hernandez and Dehghani-Sanij, 2019). However, such assistance for $S_{t}TS_{i}$ has not been well explored, despite its importance. The $S_{t}TS_{i}$ movement requires eccentric control of the knee extensors in collaboration with the hip extensors to modulate the gravitational descent of the body, while maintaining postural stability (Jeon et al., 2024). It is desirable to design and realize power assistance for both STS motions within the same framework because $S_{i}TS_{t}$ is inherently coupled with $S_{t}TS_{i}$ as these transitions typically occur as a complementary pair. It is crucial that such assistive devices activate properly and in a timely manner, as well as that they are well aligned with the user’s intent to initiate movement. If a device is incorrectly activated before the user is ready or if its activation is substantially delayed, it can result in loss of body balance or excessive joint loading as well as reduced device usability.

Because $S_{i}TS_{t}$ and $S_{t}TS_{i}$ transitions are complementary actions that frequently occur in daily life, it is desirable to develop a unified system capable of providing power assistance for both within an integrated control framework. Another important consideration is the incorporation of common STS motion strategies that are naturally adopted in everyday movements. Moreover, we aim to achieve a concise representation and analysis of muscle collaborations by introducing muscle synergy analysis that provides widely accepted representation of muscle coordination (Ting and McKay, 2007). This framework facilitates physiologically interpretable comparisons of neuromuscular control across different movement strategies.

We investigated the feasibility of temporally forecasting motion initiation using muscle synergy derived from myoelectric measurements and verified the possibility of activating assistive devices at appropriate timing during STS motions.

The primary contributions of this study are as follows:

1. Both STS motions can be sufficiently represented by only four muscle synergy patterns.
2. These four synergy patterns are common to two STS motion strategies, namely, the momentum transfer strategy and the stabilization strategy, which are commonly adopted in daily STS motions.
3. State changes during STS motions, i.e., initiation of $S_{i}TS_{t}$ and $S_{t}TS_{i}$ , can be forecasted using muscle synergy up to 300 ms prior to their occurrence with more than 90% accuracy and within a temporal error of 50 ms.

## 2 Related works

A variety of devices, such as actuated chairs (Kuroda et al., 2022; Abdul Ghani and Tokhi, 2016), walkers (Leung and Yeh, 2011; Bulea and Triolo, 2012), and robotic exoskeleton suits (Furukawa et al., 2022; Lefèvre et al., 2024), have been developed to assist STS motions. Rehabilitation environments using robotic power assistive devices have also been explored, for example, exoskeletons (Furukawa et al., 2022), which compensate for insufficient muscle strength and enable successful movement execution. Through these developments, there has been increasing demand for automatic control of such devices based on the user’s motion intention. This capability contributes to improved usability and safety and to enhancement of the user’s sense of self-agency during motion (Bortole et al., 2015).

Numerous studies, including (Carvalho et al., 2023; Wang et al., 2021), have demonstrated the effectiveness of detecting the intentions responsible for STS motions. To date, one common approach has been the detection of posture changes that correspond to the onset of predefined motions (Lu et al., 2023; Pérez-Ibarra et al., 2020). For example, at the beginning of an $S_{i}TS_{t}$ motion, the upper body leans forward to maintain the center of gravity within the base of support. The displacement of the torso can therefore be used to trigger assistive devices. However, relying solely on posture changes may cause substantial delay and misdetection of the user’s intention, leading to malfunction of assistive devices. Because posture changes are the consequence of muscle contractions during motion execution, their detection inherently introduces a delay relative to motion initiation. Such latency degrades motion smoothness and usability, as well as the user’s sense of self-agency. Detecting motion intention at the earliest possible stage is therefore critical as it provides sufficient time for assistive devices to reach full operational capacity. Furthermore, similar posture changes may occur during other actions, such as leaning forward to retrieve an item from a table or maintaining a bent posture to touch the feet. These motions may be misclassified as $S_{i}TS_{t}$ if the system relies exclusively on posture or joint angle information.

To address the above challenges, numerous studies on motion detection have employed electromyography (EMG). (Fukuda et al., 2003). showed that EMG signals exhibit favorable characteristics for controlling robotic arms, even under conditions of muscle fatigue and sweating. (Andreasen et al., 2005). developed an EMG-based system to facilitate upper-extremity rehabilitation in patients with neurological impairments. (Lenzi et al., 2012). proposed the use of EMG signals to control an elbow exoskeleton based on elbow joint movement. EMG offers several advantages, most notably its ability to reflect the level of muscle activation. As changes in myoelectric potential precede actual muscle contraction, EMG enables early detection of intended movement before it becomes externally observable (Fleming et al., 2021). In the HAL-3 system, (Kawamoto and Sankai, 2002), utilized EMG to detect lower-limb muscle activation during gait, thereby improving system responsiveness. However, the computation required for motion detection and subsequent device activation often takes longer than the physiological lead time provided by EMG signals. Typically, pneumatic assistive devices require 100 ms to deliver full output force, and EMG sensing and signal processing for device control also introduce non-negligible delays. Additionally, power-assist devices interact with the user’s body (e.g., by pushing or pulling) through soft tissues such as fat and muscle, which further delays effective force transmission to the body. These factors may result in undesirable delay and unnatural behavior in assistive devices.

Another factor that must be considered is the motion strategy used for STS, which may be arbitrarily selected by the person performing the movement. Numerous studies have examined differences in STS motions between younger and older adults, e.g., (Fujimoto and Chou, 2012; Papa and Cappozzo, 2000). In these studies, motion strategies are generally categorized into the following: a momentum transfer strategy and a stabilization strategy. Older adults often require greater stability during $S_{i}TS_{t}$ and $S_{t}TS_{i}$ transitions, achieved by lowering their center of gravity, reducing movement speed, and ensuring that it remains within their base of support (i.e., the feet). This approach is referred to as the stabilization strategy, involving slower and more segmented movement, with reduced momentum and enhanced stability for postural control throughout the task. In contrast, younger individuals tend to adopt the momentum transfer strategy, in which forward and upward momentum is generated through trunk flexion and rapid hip and knee extension during $S_{i}TS_{t}$ , and *vice versa* during $S_{t}TS_{i}$ . This strategy is generally more energy efficient and faster, but it requires rapid muscle contraction together with greater dynamic balance and coordination. Motion forecasting and assistance must therefore adapt to both strategies since either may be employed during an actual STS motion. Adapting to these two distinct strategies has remained an important unresolved issue to date.

## 3 Key idea

In this study, we focused on two main aspects:

1. Muscle synergy analysis of two STS motions ( $S_{i}TS_{t}$ and $S_{t}TS_{i}$ ) performed under two common motion strategies (the stabilization strategy and the momentum transfer strategy).
2. Motion prediction, namely, forecasting motion initiation to ensure sufficient anticipation time for activating an assistive device.

Muscle synergy is a widely accepted concept in neuromuscular control, whereby movements of the musculoskeletal system are represented by fewer coordinated activation patterns. This framework simplifies the control of the highly redundant musculoskeletal system and allows different movements to share common activation structures, thereby reducing overall system complexity. Moreover, each muscle synergy pattern is closely associated with the action of specific joints, and combinations of these patterns describe coordinated multijoint movements. Consequently, muscle synergy activation provides information that can be directly exploited for controlling assistive devices supporting joint motion. After identifying a compact set of synergy patterns that account for two STS motions executed under distinct strategies, this study investigated a unified motion forecasting framework based on these patterns. A deep neural network (DNN) was designed to forecast future motion states, specifically muscle synergy activation, and its performance was evaluated using experimental motion data.

## 4 Data collection

This study was conducted in accordance with all relevant institutional ethical guidelines and was approved by RIKEN (ethical protocol RIKEN-W1-2024-018). All data collection and analysis procedures followed approved ethical standards.

We collected data for $S_{i}TS_{t}$ and $S_{t}TS_{i}$ motions performed under two common strategies, namely, the momentum transfer strategy and the stabilization strategy, using EMG and motion capture system. Eight young, healthy male participants with an average height of 178.1 cm and an average body weight of 69.67 kg were recruited. Each participant was initially seated on a chair adjusted to maintain a knee joint angle of 90°. To ensure consistency in the sitting condition, the seat height was individually adjusted to match each participant’s knee height. Subsequently, participants were instructed to repeatedly perform a sequence consisting of sitting still, $S_{i}TS_{t}$ , standing still, and $S_{t}TS_{i}$ . During the standing and sitting states, participants maintained the posture for at least 5 s. For each strategy, stabilization and momentum transfer, participants completed 30 cycles of $S_{i}TS_{t}$ and $S_{t}TS_{i}$ motions, resulting in a total of 60 motion cycles per participant.

For EMG recording, eight muscles involved in $S_{i}TS_{t}$ and $S_{t}TS_{i}$ motions were measured, which primarily contribute to hip, knee, and ankle joint moments as well as postural stability: vastus medialis (VM), rectus femoris (RF), vastus lateralis (VL), semitendinosus (ST), tibialis anterior (TA), soleus (SOL), gluteus medius (Gmed), and erector spinae (ES) (Doorenbosch et al., 1994; Savelberg et al., 2007). EMG signals were recorded using an EMG logger (Akita et al., 2008), amplified by a factor of 1,000, high-pass-filtered with a cutoff frequency of 10 Hz, and digitized at 14-bit resolution with a sampling rate of 1 kHz. A motion capture system (OptiTrack) was used to record the positions of the shoulders, hips, knees, and ankles on the same side of the body. The recorded posture data were used solely for motion segmentation and for illustrating postures corresponding to muscle synergy peaks and were not used as input data for neural network training or prediction.

Motion states were segmented based on changes in the vertical position of the hip marker. For each STS trial, the time points corresponding to the highest and lowest hip positions were regarded as the start and end of the STS motion. The transition phases between these points were labeled as $S_{i}TS_{t}$ or $S_{t}TS_{i}$ states, depending on the direction of the height change. Consequently, the data from each participant were segmented into a repeated sequence of four states, which is denoted as Equations 1, 2:

$$S=\left\{Sitting,\;S_{i}TS_{t},\;Standing,\;S_{t}TS_{i}\right\}.$$ (1)

$$S_{X}\left(t\right)\in S,\;X\in \left\{m,s\right\}.$$ (2)

where S represents the set of motion states and t denotes time. m and s represent two distinct strategies: a momentum transfer strategy and a stabilization strategy, respectively. Muscle synergy was calculated from the recorded EMG data as follows. First, EMG signals were band-pass Butterworth filtered over 20–450 Hz with zero-phase filtering to remove bias and high-frequency noise. The filtered signals were then fully rectified (De Luca et al., 2010) and subsequently processed using a zero-phase low-pass Butterworth filter with a cutoff frequency of 4 Hz, after which muscle activation was computed.

Subsequently, a motion record is obtained as a time series of muscle activation $A^{\left(X\right)}$ as Equation 3.

$$A^{\left(X\right)}=\left\{a_{i}^{\left(X\right)}\left(t\right)\right\},\;X\in \left\{m,s,m+s\right\}$$ (3)

where $a_{i}^{\left(X\right)}\left(t\right)$ represents the muscle activation level of the i -th muscle at time t under strategy X , m and s represent a momentum transfer strategy and a stabilization strategy, respectively. m+s represents the aggregation of both strategies.

Next, non-negative matrix factorization (NMF) is performed on $A^{m}$ , $A^{s}$ and their aggregation matrix $A^{m+s}$ to extract a reduced set of muscle synergy components, as follows:

$$A^{\left(X\right)}=W^{\left(X\right)}H^{\left(X\right)}+Err,\;X\in \left\{m,s,m+s\right\}$$ (4)

where W is a matrix that represents spatial patterns. Each of its column vectors represents a time independent coordinate pattern that contains the contribution of each muscle. H is a matrix that represents temporal patterns, and each of its row vectors represents to what degree each spatial pattern is activated at each time. Error (Err) represents the residual. NMF does not produce negative elements in W and H ; this characteristic fits the nature of muscles that provide only contraction force. Moreover, to identify the number of muscle synergy patterns to fit the available data, we adopted the method of (Yang et al., 2017) to evaluate the residual |Err|(=|A−WH|) according to variance accounted for (VAF)—as implemented in (Tresch et al., 2006)—as well as the mean squared error (MSE). The minimum number n shown as Equation 5 that satisfies the following conditions was then chosen as the number of synergy patterns.

$$1\leq n\leq K,\;VAF\geq 90\%,\;MSE\leq 10^{-5}$$ (5)

where K represents the number of muscle channels, i.e., the number of measuring points. The threshold of $10^{-5}$ for MSE was determined based on the MSE calculated between the original muscle activation signals and the reconstructed signals, following the criterion reported by (Barradas et al., 2020). To obtain stable spatial patterns, concatenated non-negative matrix factorization (CNMF) as implemented in (Muceli et al., 2010) was employed. In this procedure, data from multiple trials are concatenated into A in Equation 4, which reduces the influence of trial-to-trial variability.

## 5 Muscle synergy patterns in STS motions

### 5.1 Muscle synergy patterns

**FIGURE 1:**
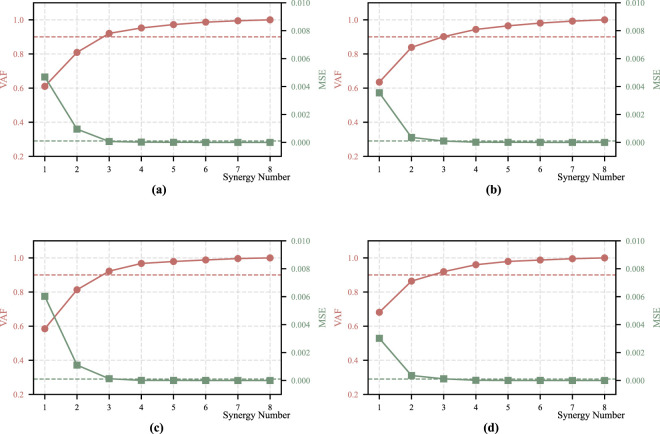
VAF and MSE quantification of each motion for Participant 1. **(a)** Momentum sit-to-stand. **(b)** Momentum stand-to-sit. **(c)** Stabilization sit-to-stand. **(d)** Stabilization stand-to-sit.

**FIGURE 2:**
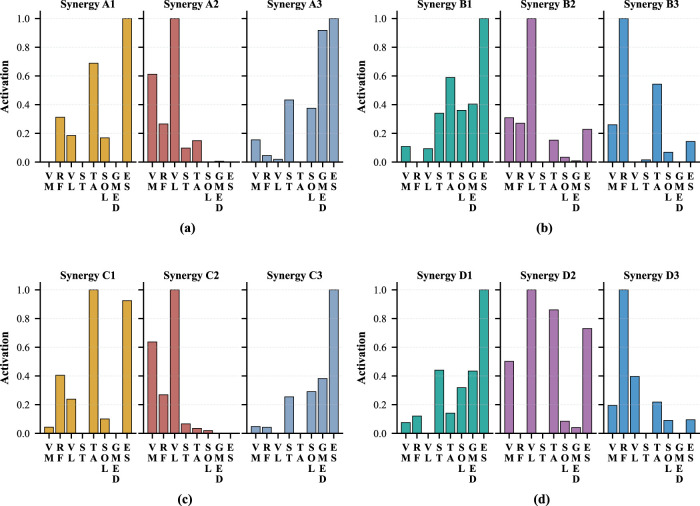
Spatial pattern of each motion under different strategies for Participant 1. **(a)** Momentum sit-to-stand. **(b)** Momentum stand-to-sit. **(c)** Stabilization sit-to-stand. **(d)** Stabilization stand-to-sit.

**FIGURE 3:**
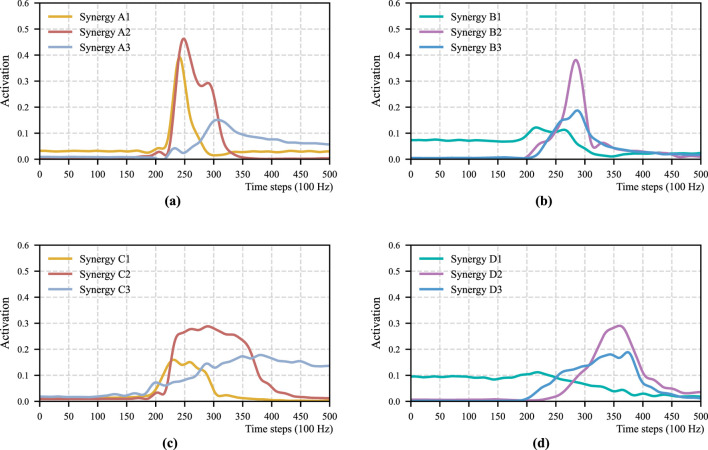
Temporal pattern of each STS motion for Participant 1. **(a)** Momentum sit-to-stand. **(b)** Momentum stand-to-sit. **(c)** Stabilization sit-to-stand. **(d)** Stabilization stand-to-sit.

CNMF was applied to the collected datasets, i.e., $A_{\text{stand}}^{m}$ , $A_{\text{sit}}^{m}$ , $A_{\text{stand}}^{s}$ , and $A_{\text{sit}}^{s}$ . Muscle synergy patterns, $W_{M}^{X},\;X\in \left\{m,s,m+s\right\},\;M\in \left\{stand,sit,stand+sit\right\}$ were obtained from the corresponding aggregated matrices $A_{M}^{X}$ . For all participants, the number of synergy patterns was determined independently based on the criteria described above, resulting in a consistent selection of three synergy patterns for both strategies of $S_{i}TS_{t}$ motions. Figure 1 shows an example from one participant illustrating the VAF and MSE values obtained with different numbers of synergy patterns. Note that this number (i.e., three synergy patterns) is consistent with previous studies reporting four synergy patterns, including (Yang et al., 2017; Yang et al., 2019). The eight muscles measured in this study do not dominantly contribute to upper-body forward leaning, which accounts for the additional synergy pattern reported in those works. Figure 2 presents the spatial patterns of the two motions under the two strategies separately. Figure 2a, A1–A3, shows the spatial patterns of the $S_{i}TS_{t}$ motion under the momentum transfer strategy, $W_{\text{stand}}^{m}$ . Similarly, Figure 2b, B1–B3, Figure 2c, C1–C3, and Figure 2d, D1–D3 shows the spatial patterns $W_{\text{sit}}^{m}$ ,the spatial patterns of $W_{\text{stand}}^{s}$ , and the spatial patterns $W_{\text{sit}}^{s}$ . Results for the remaining participants are provided in the Supplementary Material. Figure 3 shows the temporal patterns that are corresponding to the spatial patterns in Figure 2, where (a), (b), (c), and (d) show $H_{\text{stand}}^{m}\;H_{\text{sit}}^{m}$ , $H_{\text{stand}}^{s}$ , and $H_{\text{sit}}^{s}$ , respectively.

To compare the STS motions across different strategies, this study focuses on two principal aspects.

1. Cosine similarity between spatial patterns is used to quantify similarities in the relative contributions of individual muscles, thereby enabling sensitive assessment of differences in muscle coordination across motions and strategies.
2. Temporal patterns describe the sequence of muscle activations over time.

**FIGURE 4:**
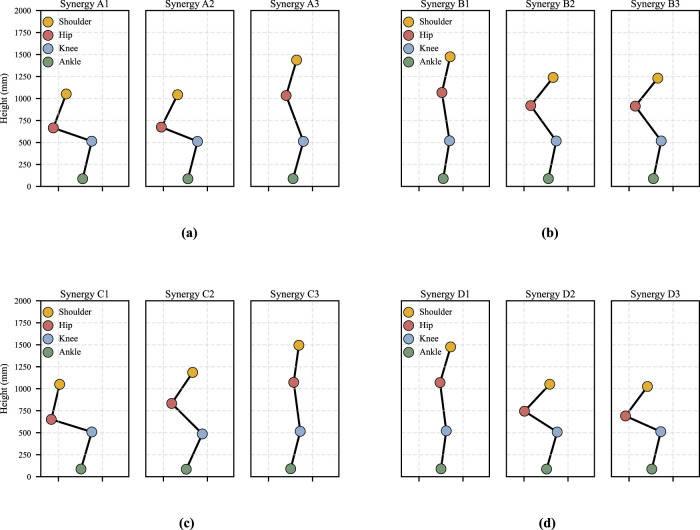
Representative postural configurations of STS motions at the peak activation of individual muscle synergies for Participant 1. Each panel depicts the posture at the peak time of the corresponding muscle synergy, represented by the location of the shoulder, hip, knee, and ankle joints. **(a)** Momentum Sit-to-stand. **(b)** Momentum Stand-to-sit. **(c)** Stabilization Sit-to-stand. **(d)** Stabilization Stand-to-sit.

The postures associated with activation peaks, together with the shapes of the temporal patterns, reflect the evolution of body configuration during movement and allow direct comparison across motions and strategies. These postures, hereafter referred to as representative postures, are illustrated in Figure 4; for example, panel (a), shows body postures corresponding to the peak timing of temporal patterns Synergy A1–A3 $\left(H_{\text{stand}}^{m}\right)$ .

#### 5.1.1 $S_{i}TS_{t}$ motions

Based on the spatial and temporal patterns observed under the momentum transfer strategy, muscle synergy patterns are characterized as follows.

Synergy A1: during the early phase of $S_{i}TS_{t}$ , TA, RF, and ES facilitate forward trunk inclination and upward movement of the upper body while maintaining balance. This synergy can be interpreted as primarily associated with ankle dorsiflexion.
Synergy A2: in the subsequent phase of $S_{i}TS_{t}$ , the VM, RF, and VL muscles generate sufficient force to achieve knee joint extension, and this synergy is, therefore, regarded as the knee extension synergy.
Synergy A3: in the later phase of $S_{i}TS_{t}$ , ST, SOL, Gmed, and ES, collectively generate force to sustain hip joint extension and maintain postural stability. This synergy can be interpreted as representing hip extension accompanied by ankle plantarflexion.

Muscle synergy patterns and corresponding postures in $S_{i}TS_{t}$ under the stabilization strategy $\left(W_{\text{stand}}^{s},H_{\text{stand}}^{s}\right)$ exhibit characteristics that are largely similar to, but subtly different from, those observed under the momentum transfer strategy. First, we can observe similar muscle coordination patterns can be identified in Figure 2: Synergy C1 functions as an ankle dorsiflexor, corresponding to A1; Synergy C2 functions as a knee extensor, corresponding to A2; and Synergy C3 functions as a hip extensor, corresponding to A3. This correspondence is supported by the cosine similarity analysis shown in Table 1, where similarities between $W_{\text{stand}}^{m}$ , $W_{\text{stand}}^{s}$ are calculated across all participants. The results indicate generally high similarity values, with the exception of one case involving A3 and C3 for participant 8, which is discussed later. In contrast to the spatial patterns, the temporal patterns under the stabilization strategy differ moderately from those of the momentum transfer strategy and are characterized by lower peak amplitudes with more prolonged activation. Because the stabilization strategy emphasizes slower and more controlled movement execution, the duration of muscle activation is correspondingly extended. Specifically, Synergy C1 and C2 exhibit lower peak amplitudes and longer activation durations than Synergy A1 and A2, respectively. In addition, the stabilization strategy requires increased muscular effort to maintain postural control, resulting in slightly greater activation of Synergy C3 relative to Synergy A3 over an extended period. The postures shown in Figures 4a,c illustrate these differences around the activation peaks of Synergy C1 and C2. In particular, the peak of Synergy C2 occurs after that of Synergy C1, which corresponds to ankle dorsiflexion. This temporal shift is consistent with the slower execution and prolonged knee extension characteristics of the stabilization strategy. Therefore, despite differences in temporal activation profiles, the two strategies in $S_{i}TS_{t}$ share the same underlying synergy structure, with Synergy A1, A2, and A3 corresponding to Synergy C1, C2, and C3, respectively.

**TABLE 1:** Cosine similarity of STS motions between different strategies. Each tuple represents cosine similarity between (Sim (A1, C1), Sim (A2, C2), and Sim (A3, C3)) in the left column, and (Sim (B1, D1), Sim (B2, D2), and Sim (B3, D3)) in the right column, where Sim (X, Y) denotes the cosine similarity between X and Y.

Participant no.	M– $S_{i}TS_{t}$ vs. S– $S_{i}TS_{t}$	M– $S_{t}TS_{i}$ vs. S– $S_{t}TS_{i}$
1	(0.98, 1.00, 0.93)	(0.99, 0.99, 0.98)
2	(0.93, 0.91, 0.84)	(0.99, 0.97, 0.95)
3	(0.99, 0.98, 0.98)	(0.98, 0.96, 0.84)
4	(0.99, 0.95, 0.73)	(1.00, 1.00, 0.98)
5	(0.96, 0.89, 0.77)	(0.98, 0.97, 0.72)
6	(0.99, 0.96, 0.94)	(1.00, 1.00, 0.94)
7	(0.99, 0.98, 0.97)	(0.95, 0.88, 0.27)
8	(0.94, 0.74, 0.38)	(0.98, 0.92, 0.28)

#### 5.1.2 $S_{t}TS_{i}$ motions

Muscle synergy patterns in $S_{t}TS_{i}$ motions were analyzed in the same manner as those in $S_{i}TS_{t}$ . First, the functional roles of the synergy patterns were examined based on $W_{\text{sit}}^{m}$ and $H_{\text{sit}}^{m}$ . During $S_{t}TS_{i}$ motion, gravity facilitates downward movement, and the primary role of the muscles is therefore to decelerate the motion while maintaining postural stability against gravitational forces. Based on the spatial and temporal patterns observed under the momentum transfer strategy, the muscle synergy patterns for $S_{t}TS_{i}$ are characterized as follows.

Synergy B1: In the early phase of $S_{t}TS_{i}$ , ST, SOL, Gmed, and ES generate force to control forward trunk motion and hip flexion. This synergy plays a central role in trunk stabilization and postural control, exhibiting relatively prolonged and increased activation to ensure controlled descent and balance. It can be regarded as a hip extensor synergy that resists gravity while coordinating ankle, knee, and hip joint torques to maintain stability.
Synergy B2: VM, RF, and VL generate forces to suppress rapid knee flexion during downward hip motion, whereas SOL contributes to suppressing excessive ankle dorsiflexion. This coordinated muscle activity provides knee extensor torque that slows the movement by resisting gravity.
Synergy B3: In the later phase of $S_{t}TS_{i}$ , TA together with VM, RF, and ES contributes to maintaining body balance while the upper body extends and inclines forward as the hips descend toward the seat. This synergy can be interpreted as an ankle plantarflexor synergy that resists gravity while supporting postural balance.

From the comparison between $W_{\text{sit}}^{m}$ (Synergy B1–B3) and $W_{\text{sit}}^{s}$ (Synergy D1–D3) in Figure 2, we observed that the two strategies exhibit similar muscle coordination patterns, although the amplitudes of individual muscle contributions differ. Specifically, Synergy B1 and D1 correspond to hip extensor function, Synergy B2 and D2 correspond to knee extensor function, and Synergy B3 and D3 correspond to ankle plantarflexion associated with maintenance of body balance. Because the VL and RF are both knee extensor muscles and can act in a complementary or alternating manner during knee extension, the spatial patterns of Synergy B2 and D2 are comparable; a similar relationship also holds for Synergy B3 and D3. The cosine similarity values shown in Table 1 support these observations, with generally high similarity across participants, except for a small number of cases, as also observed for the $S_{i}TS_{t}$ condition, despite clear differences in muscle activation amplitudes. These exceptional cases are discussed in a later section.

With respect to temporal patterns, the differences between the two strategies are similar to those observed for $S_{i}TS_{t}$ . Compared with Synergy B1–B3 under the momentum transfer strategy, Synergy D1 and D2 under the stabilization strategy exhibit lower peak amplitudes and longer activation durations, whereas Synergy D3 shows a comparable amplitude but with prolonged activation and no pronounced peak. The postures shown in Figure 4b and (d) indicate that the activation peaks of Synergy D1–D3 occur later than those of Synergy B1–B3. These characteristics are consistent with the slower execution inherent to the stabilization strategy. Therefore, despite differences in temporal activation profiles, the two strategies in $S_{t}TS_{i}$ share the same underlying synergy patterns represented by Synergy B1, B2, and B3.

### 5.2 Comparison of two STS motions

Despite their opposite directions, both $S_{i}TS_{t}$ and $S_{t}TS_{i}$ transitions require resistance to gravitational forces while maintaining postural equilibrium, with joint torques predominantly generated at the same joints. Based on this observation, we examined the feasibility of representing both STS motions using a common set of muscle synergies. To this end, cosine similarity was calculated to compare the spatial patterns of their synergies (Synergy A1–A3 and Synergy B1–B3). The results are shown in Table 2 and indicate that two pairs of spatial patterns, namely, Synergy A3 and B1, and Synergy A2 and B2, exhibit high similarity. This finding is reasonable because Synergy A3 and B1 both represent hip extensor function accompanied by ankle plantarflexion, whereas Synergy A2 and B2 represent knee extensor function, despite the opposite directions of motion. In contrast, neither Synergy A1 nor Synergy B3 has a corresponding synergy pattern in the opposite motion. Therefore, we hypothesize that a set of four muscle synergy patterns is required to adequately represent both STS motions.

**TABLE 2:** Cosine similarity between $S_{i}TS_{t}$ and $S_{t}TS_{i}$ under the same strategy. Columns under momentum correspond to (A3, B1) (A2, B2), and (A1, B3). Columns under stabilization correspond to (C3, D1) (C2, D2), and (C1, D3).

Participant	M- $S_{i}TS_{t}$ vs. M- $S_{t}TS_{i}$			S- $S_{i}TS_{t}$ vs. S- $S_{t}TS_{i}$		
	A3–B1	A2–B2	A1–B3	C3–D1	C2–D2	C1–D3
1	0.95	0.90	0.85	0.98	0.80	0.60
2	0.93	0.91	0.85	0.99	0.97	0.92
3	0.98	0.99	0.94	0.96	0.98	0.83
4	0.95	0.99	0.73	1.00	1.00	0.92
5	0.96	0.89	0.77	0.98	0.97	0.72
6	0.91	0.99	0.96	0.91	1.00	1.00
7	0.99	0.98	0.89	0.95	0.88	0.27
8	0.94	0.74	0.38	0.98	0.92	0.28
Average	0.95±0.03	0.93±0.09	0.80±0.18	0.97±0.03	0.94±0.07	0.70±0.26

### 5.3 Integration of all STS motions

**FIGURE 5:**
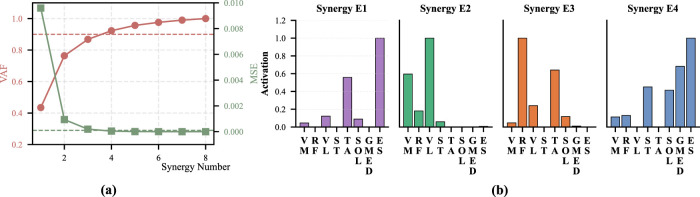
VAF-MSE quantification and spatial patterns of aggregated motions for Participant 1. **(a)** Results of VAF and MSE. **(b)** Spatial patterns of aggregated data.

Based on the discussion in the previous section, the high similarity of muscle synergies between $S_{i}TS_{t}$ and $S_{t}TS_{i}$ motions under the two strategies suggests the feasibility of integrating these motions within a unified representation. To examine the effect of such integration, muscle synergy analysis was performed on concatenated datasets and compared with the synergies obtained for each motion under a single strategy. Figure 5a presents the corresponding VAF and MSE results. These results indicate that four synergy patterns are required to represent the aggregated STS data because three synergy patterns do not satisfy the MSE criterion. Figure 5b shows the spatial patterns obtained from the aggregated data. These spatial patterns closely resemble those identified for each motion under a single strategy. Specifically, Synergy E1 shows a similarity to A1 and B3, Synergy E2 to A2 and B2, Synergy E3 to B3, and Synergy E4 to A3 and B1.

To validate this correspondence, cosine similarity was calculated between the spatial patterns of each single STS motion under a single strategy and those of the aggregated motions. Table 3 summarizes the results, where each value represents the similarity between a spatial pattern from a single STS motion ( $W_{\text{stand}}^{m}$ , $W_{\text{sit}}^{m}$ , $W_{\text{stand}}^{s}$ , and $W_{\text{sit}}^{s}$ ) and a spatial pattern from the aggregated dataset ( $W_{\text{stand}+\text{sit}}^{m+s}$ ).

**TABLE 3:** Cosine similarity between each STS motion and the aggregated data for Participant 1.

​	​	$W_{\text{STS}}^{m+s}$			
Motion state	Synergy	Cosine similarity with E1	Cosine similarity with E2	Cosine similarity with E3	Cosine similarity with E4
$W_{\text{stand}}^{m}$	A1	**0.96**	0.16	0.52	0.64
	A2	0.18	**0.99**	0.36	0.09
	A3	0.61	0.08	0.06	**0.99**
$W_{\text{sit}}^{m}$	B1	**0.89**	0.12	0.28	0.87
	B2	0.37	**0.93**	0.44	0.21
	B3	0.34	0.25	**0.95**	0.21
$W_{\text{stand}}^{s}$	C1	**0.91**	0.18	0.63	0.52
	C2	0.13	**1.00**	0.32	0.09
	C3	0.78	0.04	0.06	**0.97**
$W_{\text{sit}}^{s}$	D1	0.78	0.06	0.17	**0.98**
	D2	0.22	0.53	**0.90**	0.18
	D3	**0.75**	0.69	0.38	0.39

The bold values indicate the matched positions and corresponding values between muscle synergies A1–D3 and muscle synergies E1–E4, respectively.

Overall, our results show that each spatial pattern derived from a single STS motion under a single strategy has at least one corresponding spatial pattern among those obtained from the aggregated data. Specifically, A1–A3, B1–B3, C1–C3, and D1 exhibit high similarity to their corresponding patterns among E1–E4, as conjectured above. In contrast, the similarity values for the pairs (D2, E3) and (D3, E1) are higher than those for the expected pairs (D2, E2) and (D3, E3). This discrepancy arises primarily from the interchangeable contributions of the VM, RF, and VL, which act complementarily as knee extensors. As a result, the spatial pattern associated with knee extension may show higher similarity to an alternative synergy pattern than to the expected one. Nevertheless, for each motion under a single strategy, at least three synergy patterns exhibit high similarity to those obtained from the aggregated dataset. These findings support the hypothesis that the four spatial patterns of $W_{\text{stand}+\text{sit}}^{m+s}$ are sufficient to represent the spatial characteristics of two STS motions under two strategies.

**FIGURE 6:**
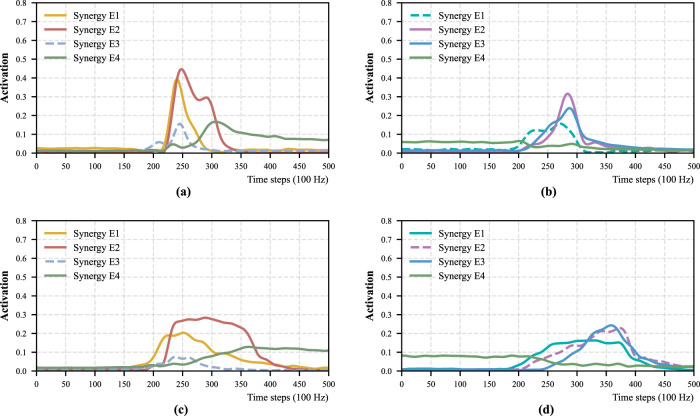
Temporal pattern of aggregated motions for Participant 1. **(a)** Momentum sit-to-stand. **(b)** Momentum stand-to-sit. **(c)** Stabilization sit-to-stand. **(d)** Stabilization stand-to-sit.

Next, we examined whether this integration adversely affects the temporal patterns when the four representative spatial patterns are used. Figure 6 shows the temporal patterns that are obtained by using $W_{\text{stand}+\text{sit}}^{m+s}$ and the following calculation:

$${\hat{H}}_{M}^{X}\left(t\right)=A_{M}^{X}\left(t\right)\;\backslash \;W_{\text{stand}+\text{sit}}^{m+s}$$ (6)

where $A_{M}^{X}$ represents motion data M∈{stand, sit} under strategy X∈{m,s,m+s} , and ${\hat{H}}_{M}^{X}$ represents the corresponding pseudo temporal patterns, and the operator “ \ ” denotes the least-squares computation used to solve the linear equation. $W_{\text{stand}+\text{sit}}^{m+s}$ denotes the above spatial patterns.

By comparing Figures 3, 6, it can be observed that the activation sequences of the temporal patterns are not substantially altered under either the momentum transfer or stabilization strategies. Note that the NMF formulation retains one degree of freedom that affects the absolute amplitude of each temporal pattern; therefore, only the temporal shape of the activation profiles is considered here. More specifically, in Figures 6a,c, Synergy E3 has no corresponding synergy pattern in $S_{i}TS_{t}$ , and it is not required to represent the original muscle activation. Similarly, Synergy E1 in Figure 6b and Synergy E2 in Figure 6d are also negligible, having no corresponding patterns in the spatial pattern set to sufficiently represent $S_{t}TS_{i}$ . Based on these observations, we conclude that a unified framework incorporating $S_{i}TS_{t}$ and $S_{t}TS_{i}$ movements performed under two different strategies can be effectively represented using four muscle synergies.

### 5.4 Analysis of infrequent cases

**FIGURE 7:**
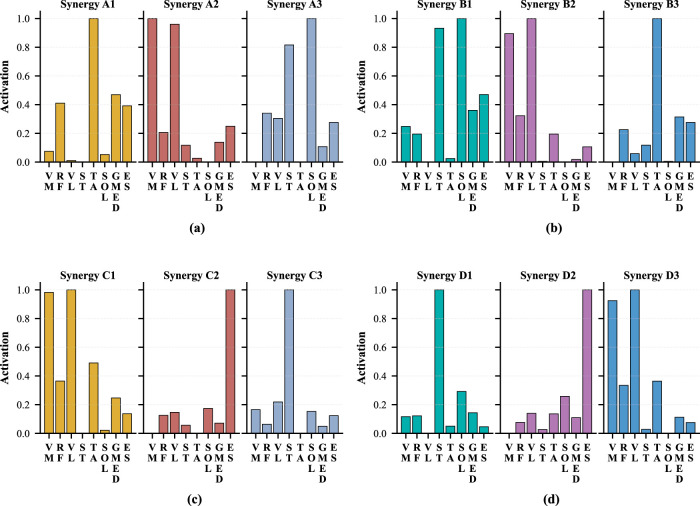
Spatial patterns of an infrequent case (Participant 8). **(a)** Momentum sit-to-stand. **(b)** Momentum stand-to-sit. **(c)** Stabilization sit-to-stand. **(d)** Stabilization stand-to-sit.

**FIGURE 8:**
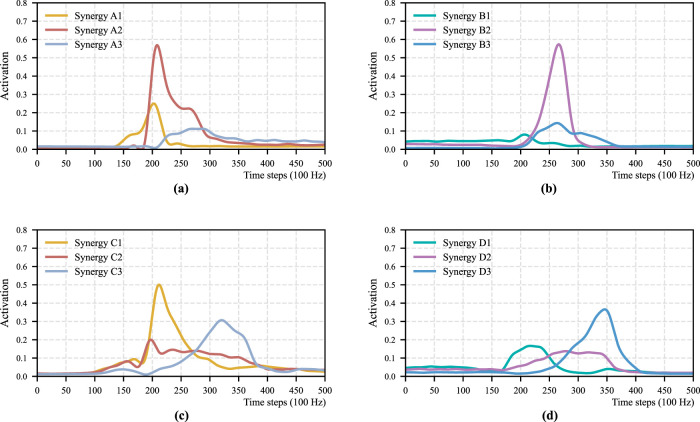
Temporal patterns of an infrequent case (Participant 8). **(a)** Momentum sit-to-stand. **(b)** Momentum stand-to-sit. **(c)** Stabilization sit-to-stand. **(d)** Stabilization stand-to-sit.

**FIGURE 9:**
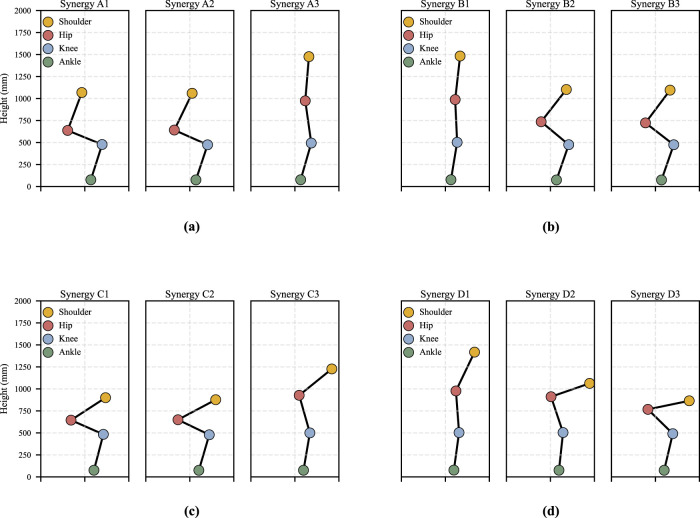
Postures of STS motions in an infrequent case for Participant 8. Each panel depicts the posture at the peak time of the corresponding muscle synergy, represented by the location of the shoulder, hip, knee, and ankle joints. **(a)** Momentum Sit-to-stand. **(b)** Momentum Stand-to-sit. **(c)** Stabilization Sit-to-stand. **(d)** Stabilization Stand-to-sit.

Here, we examine infrequent cases observed in participants seven and eight for both motions under the stabilization strategy. The corresponding spatial patterns, temporal patterns, and postures at the peak of each synergy pattern are shown in Figures 7–9, respectively. For both motions performed under the stabilization strategy, these participants tilt their trunk forward at an earlier stage, as shown in Figure 9, and subsequently maintain a nearly stationary trunk posture during the remainder of the motion. Such behavior is rarely observed in STS motions of young adults; however, it may represent a characteristic movement pattern commonly adopted by elderly individuals.

In $S_{i}TS_{t}$ under the stabilization strategy, knee extension driven by Synergy C1 the forward leaning of the trunk, which corresponds to Synergy A2 in $W_{\text{stand}}^{m}$ . During this phase, non-negligible ankle dorsiflexion torque is not required, and consequently no distinct synergy pattern corresponding to Synergy A1 emerges. To maintain the forward-leaning posture, sustained activation of the ES is necessary, which gives rise to Synergy C2 and does not have a corresponding pattern in $W_{\text{stand}}^{m}$ . In the final phase of $S_{i}TS_{t}$ , ankle plantarflexion combined with hip extension becomes necessary, resulting in activation of Synergy C3, which corresponds closely to Synergy A3 in $W_{\text{stand}}^{m}$ .

In $S_{t}TS_{i}$ forward trunk inclination while maintaining balance requires the combined generation of ankle plantarflexion and knee extension torques, producing Synergy D1, which is similar to Synergy B3. In addition, sustained activation of ES leads to Synergy D2, which resembles Synergy C2 and does not have a corresponding pattern in $W_{\text{sit}}^{m}$ . This is accompanied by activation of knee extension torque to decelerate the downward motion, represented by Synergy D3, which corresponds to Synergy B2. Although the participants’ habitual strategies reduce within-subject similarity of a given STS motion across different strategies, aggregation of the data demonstrates that four muscle synergies are sufficient to capture all four movement variants. This conclusion is supported by the results shown in Tables 3 and 4.

**TABLE 4:** Maximum cosine similarity of spatial patterns between each individual motion and the aggregated motions for each participant.

Synergy	Participant 1	Participant 2	Participant 3	Participant 4	Participant 5	Participant 6	Participant 7	Participant 8	Dominant aggregated synergy
A1	E2 (0.89)	E1 (0.97)	E1 (0.97)	E1 (0.94)	E2 (0.79)	E1 (0.97)	E4 (0.87)	E1 (0.96)	E1 (E2)
A2	E2 (0.98)	E2 (1.00)	E3 (0.98)	E2 (0.99)	E3 (0.93)	E2 (0.96)	E3 (0.99)	E2 (0.99)	E2 (E3)
A3	E3 (0.74)	E3 (0.92)	E4 (1.00)	E4 (0.92)	E4 (0.87)	E4 (0.81)	E1 (0.96)	E4 (0.99)	E4 (E3)
B1	E4 (0.82)	E3 (0.78)	E4 (0.95)	E4 (0.94)	E4 (0.96)	E4 (0.96)	E1 (0.97)	E1 (0.89)	E4 (E3)
B2	E2 (0.97)	E2 (1.00)	E3 (0.85)	E2 (0.80)	E3 (0.91)	E2 (0.94)	E3 (0.98)	E2 (0.93)	E2 (E3)
B3	E2 (0.91)	E1 (0.98)	E1 (0.99)	E1 (0.88)	E1 (0.87)	E1 (0.94)	E4 (0.86)	E3 (0.95)	E1 (E3)
C1	E1 (0.98)	E1 (0.99)	E1 (0.85)	E1 (0.95)	E1 (0.86)	E2 (0.83)	E4 (0.99)	E1 (0.91)	E1 (E2)
C2	E2 (1.00)	E2 (0.99)	E3 (0.97)	E2 (0.97)	E2 (0.87)	E1 (0.89)	E2 (0.95)	E2 (1.00)	E2 (E1, E3)
C3	E3 (0.85)	E3 (0.94)	E4 (0.99)	E3 (0.87)	E4 (0.91)	E4 (0.81)	E3 (0.96)	E4 (0.97)	E4 (E3)
D1	E4 (0.80)	E3 (0.85)	E4 (0.98)	E3 (0.98)	E4 (0.96)	E4 (0.93)	E3 (0.93)	E4 (0.98)	E4 (E3)
D2	E2 (1.00)	E2 (0.98)	E3 (0.88)	E4 (0.83)	E2 (0.88)	E2 (0.96)	E2 (0.97)	E3 (0.90)	E2 (E3)
D3	E1 (0.94)	E1 (0.97)	E1 (0.99)	E1 (0.80)	E1 (0.89)	E3 (0.90)	E4 (0.95)	E1 (0.75)	E1 (E3 ,E4)

## 6 Motion prediction

### 6.1 DNN structure

We designed a DNN to predict the future state S(t+Δt) in Equation 1 by using a sequence of temporal muscle synergy patterns H(t) , where Δt denotes the forecast horizon. Four temporal patterns are extracted from the EMG measurements and used as input to the network. The DNN is composed of the following components:

- Four Long Short-Term Memory (LSTM) layers operating at different temporal resolutions.
- A first fully connected (FC) layer to predict the future state.
- A buffer to retain short-term temporal dependencies.
- A second FC layer to enforce plausible state transitions.

**FIGURE 10:**
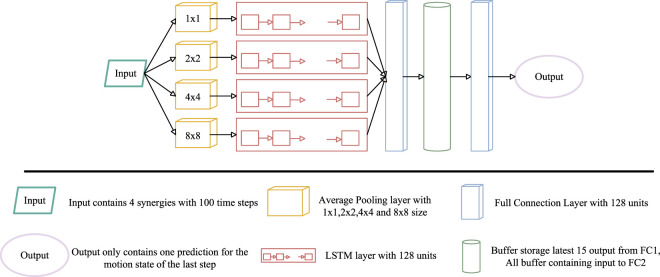
Network structure.

As shown in Figure 10, the network architecture consists of four independent LSTM layers, each with a sequence length of 100 time steps. This window covers 1.0 s for 100 Hz sampling, which provided sufficient temporal context for robust learning. In preliminary tests, shorter windows consistently led to degraded prediction performance over all participants; longer windows had not substantial improvements. Next, the input to the DNN comprises the temporal patterns after downsampling by average pooling with different strides, for example, 2, 4, and eight in our experiments. This hierarchical design enables simultaneous extraction of fine-grained temporal features and longer-term contextual information.

The final hidden states from all LSTM layers are concatenated and passed to the first FC layer, which also contains 128 units, to generate predictions of the motion state. The network is trained using the Negative Log-Likelihood Loss (NLLLoss), which is suitable for multiclass classification problems with probabilistic outputs. Training was conducted for 1,000 epochs using a batch size of 5,000. The learning rate was initialized at 0.02 and gradually decayed to 0.001 during training to promote stable convergence and mitigate overfitting.

The second FC layer is introduced to suppress unacceptable state transitions. Although infrequent, sporadic misclassifications produced by the first FC layer were found to degrade the perceived reliability of the system. For example, if a participant remains in a sitting state, predicting the subsequent state as “standing” or “stand-to-sit” represents an impossible transition. In addition to such physically implausible transitions, excessively short state durations or unrealistically rapid transitions, for example, a “sit-to-stand” state lasting only 10 ms, are also undesirable.

To exclude these invalid transitions, a second FC layer was incorporated. This layer takes as input the current prediction from the first FC layer and preceding predictions retained in the buffer, and outputs a temporally plausible state estimate. This design allows the model to incorporate short-term prediction history, thereby reducing semantically inconsistent, that is, unacceptable, errors during real-time operation. The buffer length was empirically set to 15 based on the temporal characteristics of STS motion transitions. Using a shorter buffer markedly reduced the model’s ability to suppress inconsistent predictions, whereas increasing the buffer size beyond 15 resulted in diminishing performance gains while reducing the effective forecast horizon.

### 6.2 Real-time prediction scheme

In real-time processing, the forecasting procedure must sequentially predict future states with minimal latency using only current and past inputs. To enable efficient sequential computation during real-time operation, several modifications were introduced as follows. Although the low-pass filtering approach described in Section 5.1 (4 Hz Butterworth filter) is effective for removing high-frequency components from EMG signals, it introduces substantial delay owing to phase distortion. Instead, a Kalman filter was employed for real-time processing. The parameters of the Kalman filter were determined as Equation 7:

$$\begin{array}{cc} & \left(1\right)\text{ State equation:}\;x_{t}=Gx_{t-1}+w_{t},\;w_{t}∼N\left(0,\sigma_{B}^{2}\right),\\ & \left(2\right)\text{ Observation equation:}\;y_{t}=Fx_{t}+v_{t},\;v_{t}∼N\left(0,\sigma_{V}^{2}\right). \end{array}$$ (7)

The process noise variance $\left(\sigma_{B}^{2}\right)$ was set to a small value (e.g., 0.1), thereby allowing the Kalman filter to track overall signal trends smoothly rather than overreacting to minor fluctuations. The observation noise variance $\left(\sigma_{V}^{2}\right)$ was set to a larger value (e.g., 10), which is designed to reflect the substantial noise typically present in EMG signals. This parameter setting enables the Kalman filter to produce a smooth output from highly fluctuating EMG measurements.

In muscle synergy computation, NMF in Equation 4 is performed over the entire motion sequence, which implies that W and H(t) cannot be obtained until the motion has finished. To adapt this computation to sequential input, we assume that the spatial patterns in W do not change significantly over short time intervals (Ilham et al., 2024), and can therefore be well approximated by the representative spatial patterns $W_{\text{stand}+\text{sit}}^{m+s}$ obtained from the training data, as described in Section 5.1. Accordingly, the temporal patterns H(t) are estimated sequentially using Equation 6, where $W_{\text{stand}+\text{sit}}^{m+s}$ is used as W , “strategy” includes both strategies, and “motion” includes $S_{i}TS_{t}$ and $S_{t}TS_{i}$ .

## 7 Experimental results for state prediction

### 7.1 Experimental setting

For experimentation, we first collected 60 aggregated motion trials for each participant, as described in Section 4. Using the prediction network introduced in Section 5.1, the DNN was applied to predict motion states with a specified forecasting horizon. The input to the DNN consisted of the temporal patterns H(t) , which were downsampled from the original sampling rate of 1 kHz–100 Hz. The four muscle synergy spatial patterns $\left(W_{\text{stand}+\text{sit}}^{m+s}\right)$ derived from the training data were used as representative spatial patterns. Ground-truth motion states S(t) , used for training and accuracy evaluation, were determined from the vertical displacement of the hip marker recorded by the OptiTrack motion capture system, as described in Section 4.

To evaluate the performance of motion state prediction results S(t) , statistical analyses were conducted across multiple forecast time horizons ranging from 100 to 500 ms. For cross validation, 80% of the data were randomly chosen for training, remaining 10% for validation, and 10% for testing. To avoid temporal leakage, each strategy-specific dataset was partitioned chronologically into three non-overlapping segments, and the temporal order was preserved within the training, validation, and test sets. The corresponding segments from two datasets were then concatenated to form the final training, validation, and test sets. At each forecast horizon, the model was trained and evaluated independently ten times, and the average performance was reported to reduce the effects of random initialization and training variability.

Prediction accuracy (ACC) was computed over all samples in the test set to assess the overall performance. The definition of the accuracy is given as Equation 8:

$$\text{Accuracy}=\left(\frac{\sum_{t=1}^{N}\delta \left(\hat{S}\left(t\right),S\left(t\right)\right)}{N}\right)\times 100\%$$ (8)

where N is the total number of samples, and δ(⋅,⋅) denotes the indicator (Kronecker delta) function, i.e., δ(a,b)=1 if a=b and 0 otherwise. This approach reflects the model’s ability to deliver consistent predictions throughout the full operation of the real-time system, rather than concentrating solely on discrete transition events. Considering population imbalance across classes, we additionally report class-wise precision, recall, and F1-score for each motion state class c∈S shown as Equation 9:

$$\begin{array}{cc} {\text{Precision}}_{c} & =\frac{{\text{TP}}_{c}}{{\text{TP}}_{c}+{\text{FP}}_{c}},\\ {\text{Recall}}_{c} & =\frac{{\text{TP}}_{c}}{{\text{TP}}_{c}+{\text{FN}}_{c}},\\ {\text{F}1}_{c} & =\frac{2\;{\text{Precision}}_{c}\;{\text{Recall}}_{c}}{{\text{Precision}}_{c}+{\text{Recall}}_{c}}. \end{array}$$ (9)

### 7.2 Performance evaluation

**FIGURE 11:**
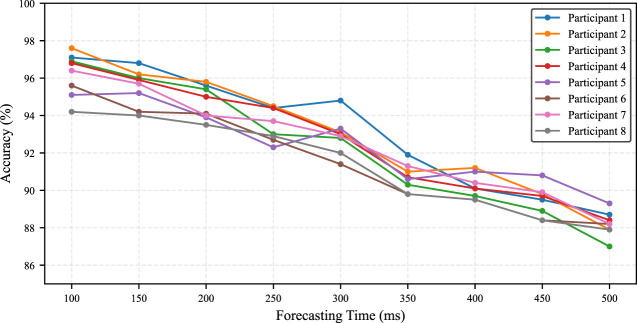
Predictive accuracy for different forecasting horizons.

As shown in Figure 11, prediction performance was evaluated at forecast horizons ranging from 100 ms to 500 ms for each participant. A forecast horizon of 300 ms was selected for further evaluation to ensure stable prediction accuracy as the model achieved a mean accuracy exceeding 90% under this condition. The network was trained to predict future states, for example S(t+300 ms) , corresponding to the motion state 300 ms ahead. Based on the observed DNN performance, the model was trained using three different data configurations: (1) momentum transfer STS data only; (2) stabilization STS data only; (3) a combination of momentum transfer and stabilization STS data. The corresponding prediction results are summarized in Table 5.

**TABLE 5:** Accuracy (%) obtained with different models and datasets. The model specifies the training data used, and the test data indicate the dataset to which each model is applied.

Test data	Model (training data)		
	Momentum	Stabilization	Both strategies
Momentum	94.01±0.82	53.97±0.75	93.91±0.80
Stabilization	77.74±0.84	95.18±0.69	91.78±0.87
Both strategies	84.82±0.75	73.37±0.81	92.97±0.86

Table 5 shows the prediction accuracy values for the momentum transfer and stabilization strategies separately, as well as the overall accuracy computed using the combined test set from both strategies. As shown in Table 5, models trained on a single strategy perform well for data under the same strategy, and the performance becomes worse for the other strategy. However, the model trained with data under both strategies is less degrated over under both strategies. To further assess the DNN’s ability to predict local motion state transitions, we calculated the temporal difference between the predicted transition time and the actual onset of movement. The results are summarized in Table 6. A positive value indicates that the predicted transition occurred later than the ground truth, whereas a negative value indicates that the prediction preceded the actual transition. The results show that, across all participants, the average temporal differences for all prediction types were within ±40 ms. In terms of the overall performance, 92.97 ±0.69 % of trials exhibited a time difference of less than 50 ms.

**TABLE 6:** Time difference between the ground truth and the predictions with 300 ms forecasting time.

Participants	M- $S_{i}TS_{t}$ (ms)	M- $S_{t}TS_{i}$ (ms)	S- $S_{i}TS_{t}$ (ms)	S- $S_{t}TS_{i}$ (ms)	Both-aggregated STS (ms)
1	−21.49	21.48	25.59	−21.74	22.48
2	16.86	19.75	−18.11	−18.36	18.56
3	−25.48	27.95	25.57	−28.29	−22.93
4	−35.87	36.19	32.61	−34.83	−31.73
5	−14.64	17.75	−20.47	−23.75	−21.58
6	−28.87	22.98	22.24	−26.88	−25.27
7	−22.26	26.28	−26.46	−22.92	−21.75
8	−18.59	19.49	21.59	−22.59	19.48
Average	−18.79±15.79	23.98±6.03	7.82±24.76	−24.92±5.03	−7.84±23.44

**FIGURE 12:**
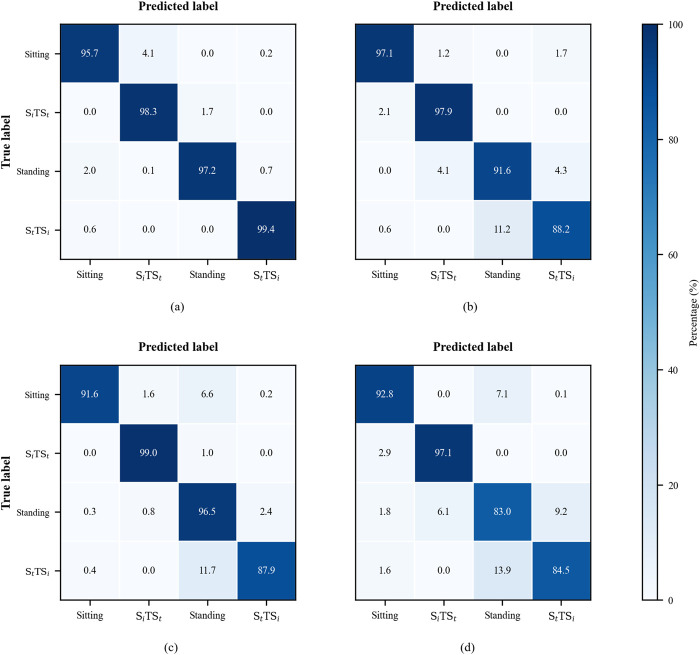
Averaged confusion matrices for the four motion classes (Sitting, $S_{i}TS_{t}$ , Standing, $S_{t}TS_{i}$ ) under the momentum and stabilization strategies. **(a)** Common case of momentum strategy. **(b)** Common case of stabilization strategy. **(c)** Infrequent case of momentum strategy. **(d)** Infrequent case of stabilization strategy.

Table 7 reports per-participant recall and F1 scores obtained with inverse-frequency class weighting. The results indicate that the proposed model achieves consistent performance across participants and motion classes. Figure 12 shows the confusion matrices computed over participants. For each participant, we first row-normalized the confusion matrix to obtain per-class percentages (rows sum to 100%), and then averaged these normalized matrices within common and infrequent cases under different strategies, separately. In Figure 12, panels (a) and (b) show the participant-averaged percentage confusion matrices for participants 1-6 (common cases), while panels (c) and (d) show the confusion matrices for participants 7-8 (infrequent cases). Figure 12 show that the most difficult portion is the prediction of $S_{t}TS_{i}$ transition under stabilization strategy for which at most 14% of transition were missed, whereas $S_{i}TS_{t}$ transitions are predicted more reliably for which less than 3% of transitions were missed.

**TABLE 7:** Per-participant recall and F1-score for the each motion states.

Participant	Recall						F1-score					
	Sitting	M- $S_{i}TS_{t}$	Standing	M- $S_{t}TS_{i}$	S- $S_{i}TS_{t}$	S- $S_{t}TS_{i}$	Sitting	M- $S_{i}TS_{t}$	Standing	M- $S_{t}TS_{i}$	S- $S_{i}TS_{t}$	S- $S_{t}TS_{i}$
P1	0.962	1.000	0.960	0.989	0.978	0.877	0.978	0.824	0.969	0.952	0.909	0.872
P2	0.926	0.935	0.976	0.876	0.917	0.932	0.960	0.731	0.980	0.768	0.901	0.913
P3	0.951	0.961	0.943	1.000	0.782	0.990	0.963	0.839	0.968	0.846	0.804	0.833
P4	0.928	1.000	0.976	0.868	0.955	0.943	0.960	0.743	0.981	0.808	0.901	0.924
P5	0.907	0.852	0.966	0.996	0.964	0.915	0.941	0.920	0.980	0.878	0.831	0.825
P6	0.972	0.986	0.901	0.893	0.959	0.902	0.970	0.833	0.940	0.750	0.940	0.813
P7	0.924	0.943	0.973	1.000	0.982	0.894	0.960	0.891	0.969	0.762	0.909	0.898
P8	0.822	0.987	0.956	0.965	0.936	0.913	0.899	0.904	0.896	0.828	0.826	0.917

### 7.3 Discussion

In this study, we confirmed that muscle synergy provides sufficient information for detecting and forecasting of two STS motions, $S_{i}TS_{t}$ and $S_{t}TS_{i}$ . We further showed that, although these motions are performed using two different strategies, they share two common synergy patterns, while each motion also exhibits one strategy-specific synergy pattern. As a result, four muscle synergy patterns are sufficient to represent both STS motions under both strategies. These findings can be explained by the underlying biomechanical characteristics of the movements.

Although $S_{i}TS_{t}$ and $S_{t}TS_{i}$ are executed in opposite directions, both motions require resistance to gravitational forces, namely, lifting the body during $S_{i}TS_{t}$ and decelerating descent during $S_{t}TS_{i}$ . Consequently, similar joint torques are required, rather than a simple reversal of joint extension and flexion. Consistent with this interpretation, inspection of the muscle synergies shown in Figure 6 and the corresponding postures in Figure 4 reveals that similar muscle groups are activated under comparable postural configurations. However, there is no direct correspondence between Synergy A1 in $S_{i}TS_{t}$ and Synergy B3 in $S_{t}TS_{i}$ . In the early phase of $S_{i}TS_{t}$ corresponding to Synergy A1, substantial joint torque is required to accelerate the movement while maintaining balance because the upper body initially has little momentum. In contrast, during the final phase of $S_{t}TS_{i}$ relatively little torque is needed to resist gravity while preserving balance. Similarly, during the phase corresponding to Synergy B3 in $S_{t}TS_{i}$ rapid reduction of momentum is required, which necessitates considerable joint torque, whereas the corresponding phase in $S_{i}TS_{t}$ does not require comparable torque because sufficient momentum has already been generated. These characteristics indicate that three of the four synergy patterns dominate within each individual STS motion.

Notably, even in the infrequent cases discussed in 5.4 in which participants employed a stabilization strategy, STS motions could still be represented using four muscle synergies. When early hip flexion is performed during $S_{i}TS_{t}$ , reduced torque is required for ankle dorsiflexion, diminishing the dominance of Synergy A1. Instead, Synergy C2, which is approximately equivalent to Synergy D2, becomes necessary to maintain forward trunk inclination. This observation further supports the conclusion that four muscle synergy patterns are sufficient to represent STS motions across both strategies.

**FIGURE 13:**
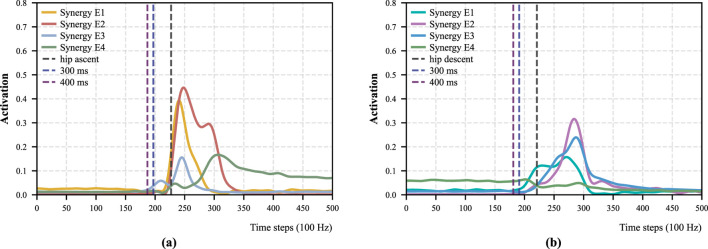
Temporal patterns of muscle synergy during STS motions under the momentum transfer strategy (Participant 1). The rightmost vertical dashed line shows the beginning of the hip ascent (or descent), and the leftmost and middle vertical dashed lines indicate different forecasting lead times (e.g., 300 ms and 400 ms) prior to hip upward. **(a)** Momentum sit-to-stand. **(b)** Momentum stand-to-sit.

With respect to the forecasting performance, our models maintained an accuracy exceeding 90% when the forecast horizon was 300 ms or less. Figure 13 illustrates the temporal relationship between the $S_{i}TS_{t}$ motion and its forecastability. Hip lift-off from the seat coincides with a rapid increase in Synergy E1 (A1) and Synergy E2 (A2), whereas subtle muscle activation corresponding to preparatory action begins approximately 500 ms before hip lift-off. For stable forecasting, it appears necessary to observe this preliminary activation until roughly 300 ms prior to hip lift-off. Consequently, when forecasting is attempted earlier than 300 ms before hip lift-off, the duration of preparatory muscle activation is insufficient to reliably predict $S_{i}TS_{t}$ .

The 300 ms horizon was not selected as an optimal window; rather, it was chosen as the maximum stable anticipation time that could be achieved while maintaining classification performance more than 90%. In our experiments, longer horizons than 300 ms led to a noticeable degradation in performance with less stability. For verifying the possibility of real-time operation, the latency of the EMG measurement through triggering of the assistive device was confirmed. The latency of EMG measurement is up to 30 ms, and preprocessing, and inference took approximately 12 ms on a PC with an NVIDIA RTX 4090. In addition, assistive devices such as pneumatic actuators typically require about 100 ms to develop full output force. Actuation for the assistive chair platform (Kogami et al., 2019; Ilham et al., 2026) requires up to 5 ms for command interface, and up to 100 ms to start elevation with sufficient force. In both cases, total latency is less than 150 ms. Thus, 300 ms prediction horizon provides sufficient margin for proactive assistive control. According to Table 6, eg., approximately 169±16 ms (= 300−150+19±16 ms) for M- $S_{i}TS_{t}$ , and 142±25 ms (= 300−150−8±25 ms) for S- $S_{i}TS_{t}$ within a one-sigma bound.

Prediction accuracy and temporal deviation exhibited different trends across motion strategies. For each strategy, when the prediction system was trained using data from the same strategy, accuracies exceeding 90% were achieved. In contrast, when the system was trained using data from a different strategy, the performance decreased; however, the accuracy remained relatively high. This result suggests that users can adopt either strategy depending on preference or situational constraints, while still benefiting from reliable motion forecasting.

Overall, the temporal difference between predicted transitions and ground truth varies across motion types and strategies. As shown in Table 5, $S_{t}TS_{i}$ performed using the momentum transfer strategy exhibited larger prediction delays than other motion types, whereas $S_{i}TS_{t}$ performed under the stabilization strategy showed greater temporal variability across participants. These differences can be explained by the intrinsic characteristics of the motions. Under the momentum transfer strategy, only brief preliminary muscle activation occurs before the onset of major activation, as illustrated in Figure 3b, which limits the available information for early prediction. In contrast, under the stabilization strategy, Synergy D2 and Synergy D3 are activated earlier, facilitating more reliable forecasting. However, during stabilization $S_{i}TS_{t}$ , the center of mass remains largely above the base of support, meaning that postural balance is well maintained. As a result, the timing and speed of movement initiation are more flexible, leading to increased inter-individual and intertrial variability.

Considering future applications, false detection (false positive) of $S_{i}TS_{t}$ is more harmful than missing (false negative), since unexpected movements of the assistive device may cause dangerous situations. To overview this problem, we calculated the frame-wise false detection rate (FDR), which is the fraction of frames labeled as Sitting in the ground truth that are incorrectly predicted as $S_{i}TS_{t}$ . For Participant 1, we observed three frames labeled Sitting but were predicted as $S_{i}TS_{t}$ , out of 1,361 Sitting frames, yielding 0.22% of FDR. For Participant 8, we observed 0.72% of FDR (11 frames false detection out of 1,538 frames). For $S_{t}TS_{i}$ , Participant one exhibited 32 false detections out of 1889 Standing frames (FDR: 1.69%), whereas Participant eight exhibited 105 false detections out of 2,386 Standing frames (FDR: 4.40%).

Once false positive $\left(S_{i}TS_{t}\right)$ is accepted, subsequent frames are classified as the next state $\left(S_{i}TS_{t}\right)$ until Standing state is detected. This configuration makes the recognition rate in Figure 12 worse than the above rate, e.g., 4.1% misdetection of $S_{i}TS_{t}$ during Sitting under momentum transfer strategy. To reduce this kind of false positive rate, we can consider tuning of the second FC layer to severely reject such misdetection. However, this may make the true positive rate worse by rejecting true transition. A proper adjustment of this trade-off is left for future work.

One of the most important future works is to verify that the proposed framework generalizes to a broader range of populations. Although no clear evidence currently indicates systematic differences in muscle activation patterns between males and females that cannot be accommodated by the proposed DNN-based framework, this assumption should be validated using data from participants of different genders and age groups. For aging, serveral works reported that healthy older and younger adults used similar knee and trunk joint mechanics as well as muscle synergy patterns, yet older adults complete the sit-to-stand trials with greater lower extremity and low back muscle activation and they show greater individual variability (Rutherford et al., 2016; Hanawa et al., 2017; Dussault-Picard et al., 2024; Monaco et al., 2010; Lo et al., 2017). Although We expect that our DNN trained with personal EMG data sufficiently captures mechanics of elderly people similarly to youngers, sufficient verification through experiments is necessary as future work.

In the present experiments, participant postures were constrained following common practice in previous studies, with hands crossed in front of the trunk. Such STS motions represent only a subset of STS activities encountered in daily life. Therefore, the framework should be extended to encompass a wider range of STS behaviors, such as $S_{i}TS_{t}$ or $S_{t}TS_{i}$ performed while holding an armrest or leaning forward onto a table. As the diversity of motions increases, safety assessment becomes increasingly important. The system must be able to detect whether a user intends to perform a natural $S_{i}TS_{t}$ or $S_{t}TS_{i}$ motion and whether the motion can be executed safely under the current conditions. If a motion with a high risk of balance loss or falling is detected, the system should immediately suppress assistance and issue an appropriate warning. Further development of these capabilities will be addressed in future work.

The number of EMG channels is also an important factor for future applications. It becomes more practical if we can reduce the number of EMG channels (electrodes); however, reduced number of EMG channels may increase sensitivity to noise and motion artifacts and may reduce prediction robustness. Future works are necessary to explore this trade-off for identifying practical sensor sets and their performance bounds. Extending the detection pipeline to broader, cross-user generalizability remains an important challenge and a key direction for future work.

## 8 Conclusion

In this study, we analyzed $S_{i}TS_{t}$ and $S_{t}TS_{i}$ motions performed under two different strategies. We demonstrated that three muscle synergy patterns are sufficient to represent each motion, including cases in which the same motion is executed using different strategies. When both motions and both strategies are aggregated, four muscle synergies are sufficient to represent the entire set of STS motions. Importantly, even though some participants exhibited infrequent variations in their movement patterns, the required number of muscle synergy patterns remained 4. We further confirmed that a real-time system can forecast STS motions using these four muscle synergy patterns, achieving a prediction accuracy of 92.97±0.86% , with prediction errors remaining within 50 ms between the predicted transition time and the ground truth. Although motion characteristics and intersubject variability differed across participants, neither the number of required synergy patterns nor the forecasting performance was substantially affected, with prediction accuracy consistently exceeding 90%.

For future work, it is necessary to validate the proposed framework in more diverse populations, including differences in gender, age, and health condition. In addition, practical deployment requires further development of safety- and usability-oriented functions, such as evaluating the safety of power assistance for STS motions under varying environmental and physiological conditions.

## Data Availability

The raw data supporting the conclusions of this article will be made available by the authors, without undue reservation.
