# Supplementary material for: Anticipatory prediction of sit-to-stand and stand-to-sit transitions: a unified approach
Source: Front Bioeng Biotechnol. 2026 Apr 1;14:1792582. doi: 10.3389/fbioe.2026.1792582 (PMC13079333; doi:10.3389/fbioe.2026.1792582)
Supplement: Supplementary file 1 [file DataSheet1.pdf]

Supplementary Material

1 FIGURES

This supplementary material provides supplementary experimental results, including the full spatial and temporal synergy patterns for all participants. These results are consistent with the main findings reported in the manuscript.

The spatial patterns were extracted from the aggregated EMG data across motions within each movement strategy, whereas the temporal patterns represent the corresponding activation time courses for individual motions, computed as the median activation profiles across repetitions.

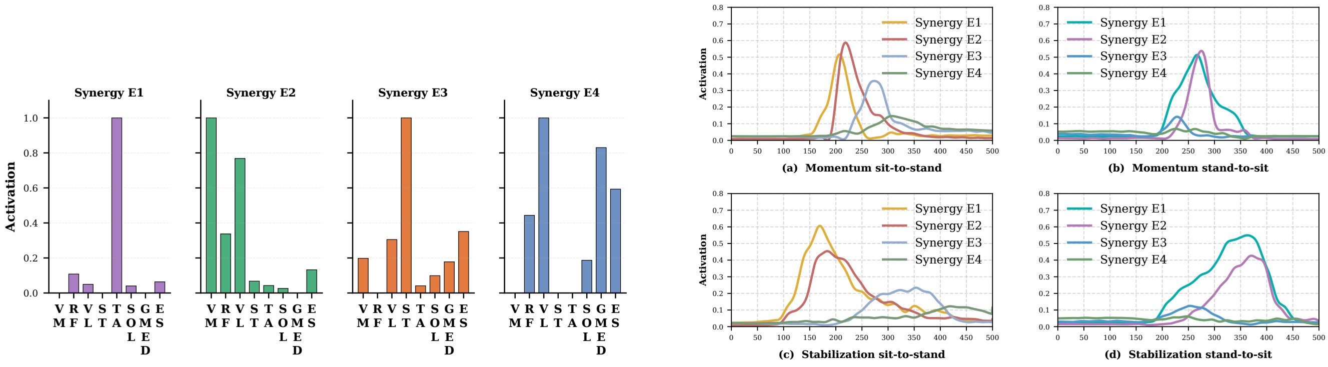

(A) (B)

Figure S1. Muscle synergy patterns of aggregated data (participant 2). **(A)** Spatial patterns. **(B)** Temporal patterns.

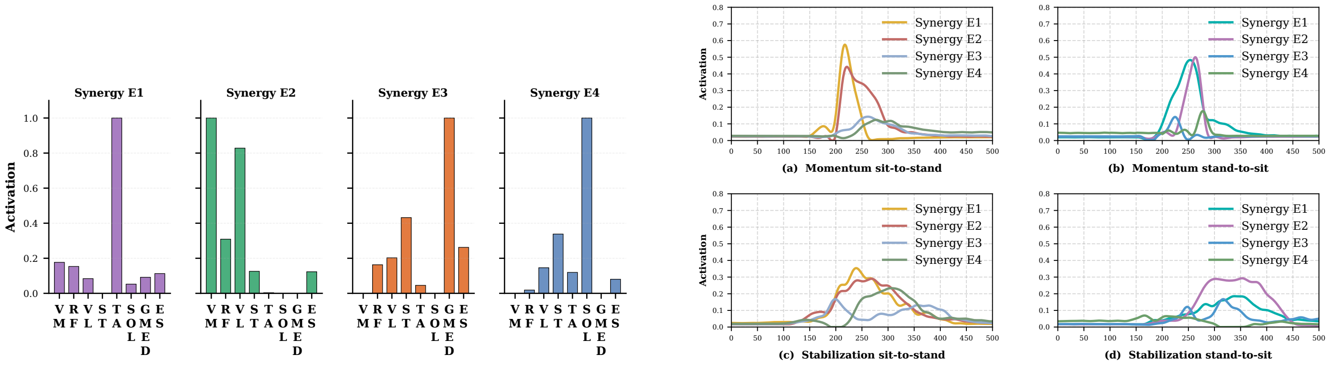

(A) (B)

Figure S2. Muscle synergy patterns of aggregated data (participant 3). **(A)** Spatial patterns. **(B)** Temporal patterns.

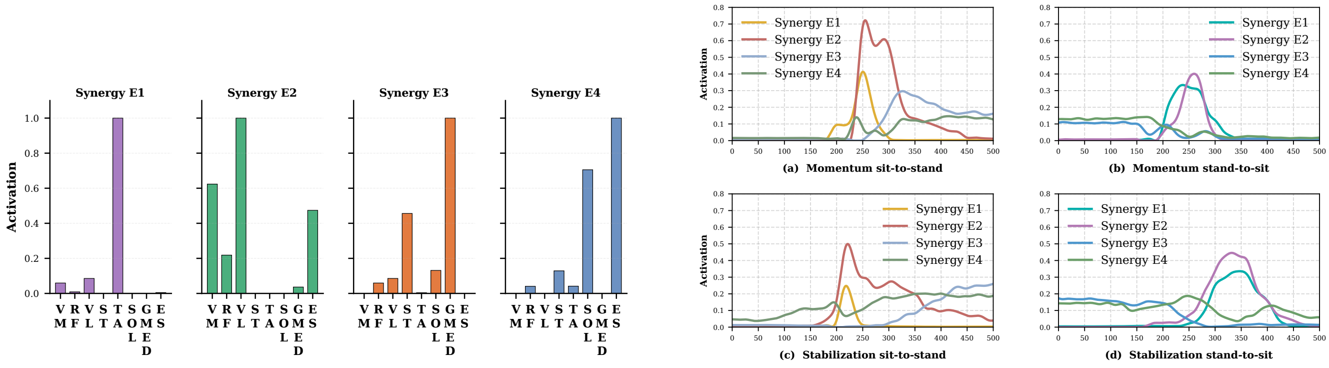

(A) (B)

Figure S3. Muscle synergy patterns of aggregated data (participant 4). **(A)** Spatial patterns. **(B)** Temporal patterns.

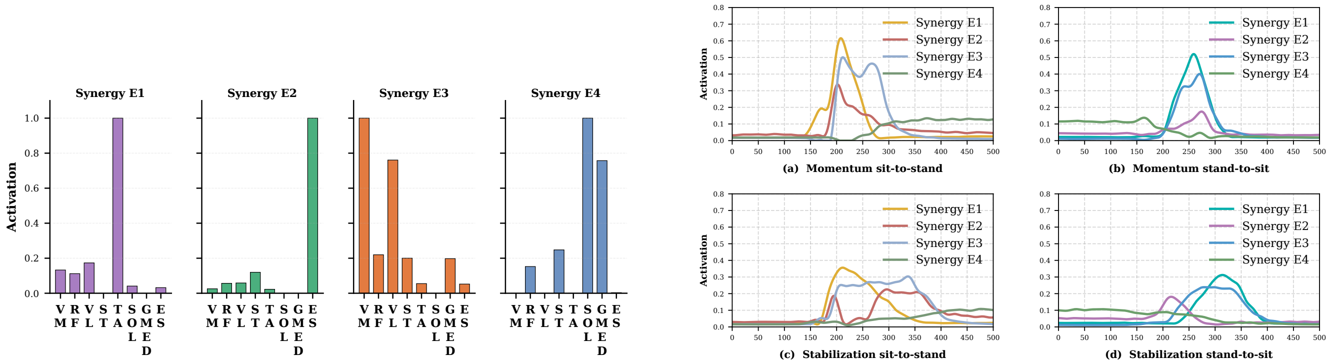

(A) (B)

Figure S4. Muscle synergy patterns of aggregated data (participant 5). **(A)** Spatial patterns. **(B)** Temporal patterns.

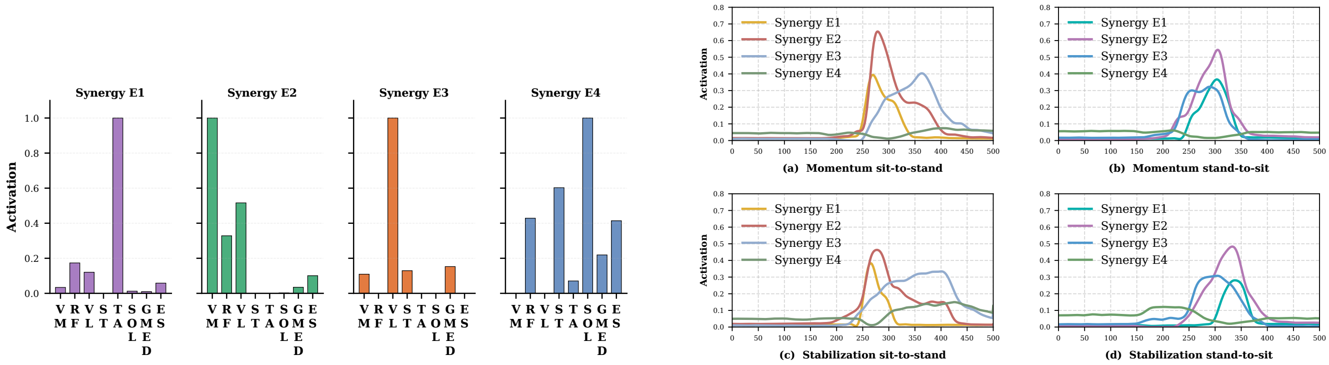

(A) (B)

Figure S5. Muscle synergy patterns of aggregated data (participant 6). **(A)** Spatial patterns. **(B)** Temporal patterns.

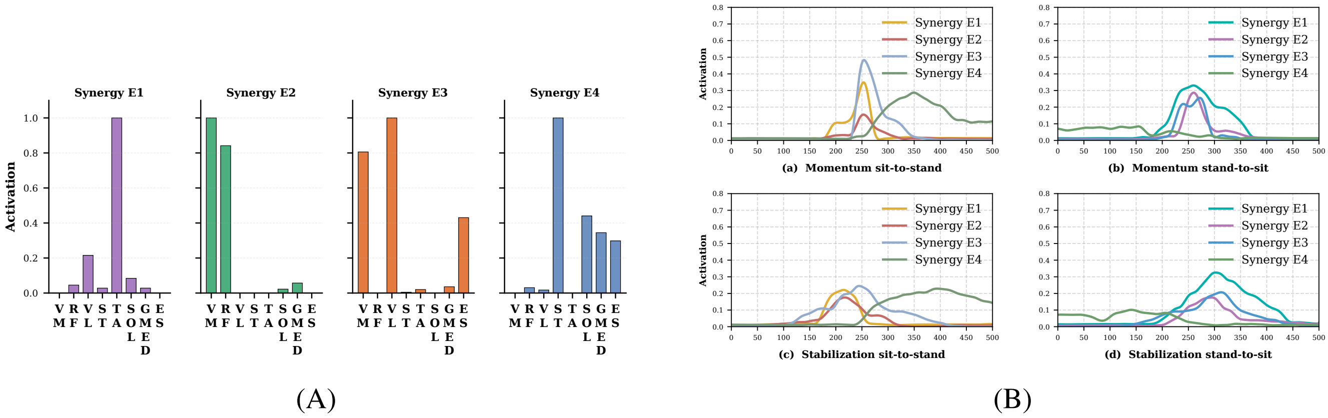

Figure S6. Muscle synergy patterns of aggregated data (participant 7). **(A)** Spatial patterns. **(B)** Temporal patterns.

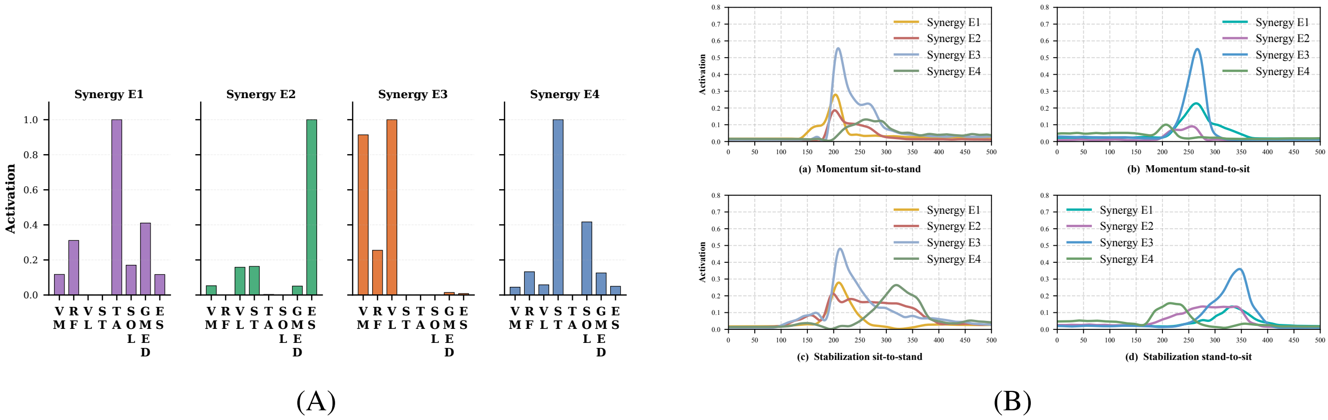

Figure S7. Muscle synergy patterns of aggregated data (participant 8). **(A)** Spatial patterns. **(B)** Temporal patterns.
